# Disparities in registration and use of an online patient portal among older adults: findings from the LitCog cohort

**DOI:** 10.1093/jamia/ocv025

**Published:** 2015-04-25

**Authors:** Samuel G Smith, Rachel O’Conor, William Aitken, Laura M Curtis, Michael S Wolf, Mita Sanghavi Goel

**Affiliations:** ^1^Division of General Internal Medicine and Geriatrics, Northwestern University, Chicago, IL, USA; ^2^Wolfson Institute of Preventive Medicine, Queen Mary University of London, London, UK; ^3^Department of Learning Sciences, Northwestern University, Evanston, IL, USA

**Keywords:** patient portal, disparities, health literacy, race, education

## Abstract

**Objective** To document disparities in registration and use of an online patient portal among older adults.

**Materials and methods** Data from 534 older adults were linked with information from the Northwestern Medicine Electronic Data Warehouse on patient portal registration and use of functions (secure messaging, prescription reauthorizations, checking test results, and monitoring vital statistics). Age, gender, race, education, self-reported chronic conditions, and the Newest Vital Sign health literacy measure were available from cohort data.

**Results** Most patients (93.4%) had a patient portal access code generated for them, and among these 57.5% registered their accounts. In multivariable analyses, White patients (*P* < .001) and college graduates were more likely to have registered their patient portal (*P* = .015). Patients with marginal (*P* = .034) or adequate (*P* < .001) health literacy were also more likely to have registered their patient portal. Among those registering their accounts, most had messaged their physician (90%), checked a test result (96%), and ordered a reauthorization (55%), but few monitored their vital statistics (11%). Adequate health literacy patients were more likely to have used the messaging function (*P* = .003) and White patients were more likely to have accessed test results (*P* = .004). Higher education was consistently associated with prescription reauthorization requests (all *P* < .05).

**Discussion** Among older American adults, there are stark health literacy, educational, and racial disparities in the registration, and subsequent use of an online patient portal. These population sub-group differences may exacerbate existing health disparities.

**Conclusions** If patient portals are implemented, intervention strategies are needed to monitor and reduce disparities in their use.

## BACKGROUND AND SIGNIFICANCE

The adoption of electronic health records (EHRs) by hospitals and providers in the United States is rising.^1^ This trend is likely to continue following the Health Information Technology for Economic and Clinical Health act, which authorized incentive payments to increase provider adoption and meaningful use of EHRs.^2^ Patient portals are secure websites for patients, typically maintained by provider practices, that offer access to a variety of functions linked to a physician’s EHRs.^3^ Most patient portals offer similar basic functions, including the ability to view protected health information (e.g., lab results, medication lists, immunizations), refill prescription medications, schedule appointments, and send secure messages to providers.^4^ Evidence from randomized trials suggests patient portals can improve health outcomes and patient satisfaction,^5–8^ but data have been inconsistent.^4^

Federal incentives for health systems and providers may not translate to increased, equitable adoption of patient portals by patients. Reports suggest ∼30–70% of eligible patients accept the offer of a patient portal.^9–12^ The differential acceptance of this technology between population sub-groups may exacerbate disparities in health outcomes. Initial reports suggest low levels of use among socio-demographic sub-groups such as racial and ethnic minorities,^11^^,^^12^ those with less education,^4^^,^^12^ and older patients.^11^^,^^13^^,^^14^ It is important to document these disparities as a first step toward preventing downstream effects on health and health care.

Older patients may be a particularly important group to study, as they are more likely to have chronic conditions, a factor which has been shown to increase uptake of patient portals.^4^ However, the usability of patient portals is a significant barrier,^9^^,^^15–18^ and this may be a particular problem for older adults less familiar with information technology. National data suggests older adults are less likely to make use of online health information, including treatment and quality comparison tools, and advice about chronic conditions and disease prevention.^19^ Older adults are likely to be the most frequent users of healthcare, but objective portal usage data among this population group are lacking.

Health literacy is an additional risk factor that may explain lower uptake and use of patient portals. The Institute of Medicine defines the construct as the “the degree to which individuals have the capacity to obtain, process, and understand basic health information and services needed to make appropriate health decisions.”^20^ A study of diabetic patients reported no relationship between self-reported health literacy and accessing a patient portal,^21^ although a larger and more comprehensive report linked low self-reported health literacy with lower levels of patient portal registration, logins, and use of patient portal functions.^22^ However, people are typically poor judges of their own abilities,^23^ and self-report literacy measures do not assess the same latent construct as objective health literacy assessments.^24^ This is supported by the low to moderate correlation that is observed when comparing objective and subjective assessments.^25^^,^^26^ Research documenting the association between objective health literacy assessments and patient portal use is needed to highlight potential disparities.

## OBJECTIVE

Limited health literacy is more prevalent among older adults,^27^ with a meta-analysis suggesting nearly 40% of adults aged over 50 have limited basic skills.^28^ As patient portal adoption and use is inversely associated with age,^11^^,^^13^^,^^14^ we investigated this topic further among a cohort of older adults who had completed objective health literacy assessments and were current patients in a health system offering patient portal access. In a two-step process using a sample of older adults, we aimed to establish whether socio-demographic factors and health literacy were associated with 1) registering for a patient portal account and 2) using the portal’s functions after registration.

## METHODS

### Sample

The sample was part of the Health Literacy and Cognitive Function among Older Adults study (also known as LitCog).^29^ Starting in 2008, LitCog participants were recruited from 1 academic general internal medicine ambulatory care clinic and from 5 federally qualified health centers in Chicago, Illinois. Electronic records were used to identify 3176 English-speaking patients aged 55–74 years of age who had at least two clinic visits within the past 18 months of the baseline LitCog interview. Of these individuals, 1904 were randomly selected and notified of the study by mail, and patients were able to opt out at this stage. After screening by telephone, 244 people were excluded due to cognitive and hearing impairment, limited English proficiency, and lack of affiliation with a clinic physician (i.e., less than two recorded visits in the previous 2 years). A total of 794 people refused, 20 were eligible but had scheduling conflicts, 14 were deceased, and 4 were duplicate records. The final sample included 828 participants for a cooperation rate of 51% following American Association for Public Opinion Research guidelines.^30^ Only patients recruited from the general internal medicine ambulatory care clinic were used in this study (*n* = 628), as the remaining patients were identified through 1 of 5 federally qualified health centers where a portal was not available to patients. Of the remaining 628 patients, 94 had not visited the hospital and had not logged into the patient portal in the 2 years prior to the extraction of the portal use data (May 2014). They were therefore excluded, leaving a final sample of 534 patients.

### Context

The internal medicine clinic was based at Northwestern Medical Faculty Foundation, the group practice for faculty of the Feinberg School of Medicine of Northwestern University. The commercial EHR used is EpicCare (version Spring 2007, Epic Systems Corporation). Starting in March 2006, physicians were encouraged to offer access to patient portals, but it was at their discretion and they could opt not to offer activation codes. The process for registering the patient portal required that: 1) an access code was generated by the provider and given to the patient; 2) patients needed to log onto a designated website on their own desktop or tablet device, or download an app onto their mobile device; 3) patients entered their personal registration code and other identifying information; and 4) patients created a unique user name and password. After completing the registration process and logging in, the home screen presents patients with three main options (message a provider, request a prescription reauthorization, and view test results). On the same screen, a side bar presents additional options including personal health records (monitoring vital statistics [e.g. height, weight, body mass index, body surface area, blood pressure, heart rate, breathing rate, temperature], previous conditions, and current conditions), previous or upcoming appointments, sent and received messages, personal profile, and a help page. A link to an introductory video is available on the home page. Once an account is registered, an option is available to cancel the account, should the patient choose to.

### Data collection

Between August 2008 and October 2011, the LitCog cohort completed 2 face-to-face baseline interviews, 7–10 days apart. Two interviews were necessary to limit patient fatigue. Only data from the day-one interview are used here, because data collected during the day-two interview were unrelated to this study’s objectives. A research assistant administered a battery of measures, including health literacy and socio-demographic assessments. Using the unique patient hospital identification numbers, data from the LitCog cohort were linked to patient portal usage data recorded by the Enterprise Data Warehouse (EDW) between March, 2006 and May 21, 2014. The EDW consolidates clinical and biomedical data from all patients receiving care at Northwestern Medicine for the use of quality improvement and research purposes. For this study, data was restricted to the general internal medicine ambulatory care clinic outpatient data for patient portal use in relation to an outpatient physician. This is because data for the federally qualified health centers was not available and inpatient services at this institution are not linked to the patient portal.

### Measures

#### Patient portal registration and usage

The generation of a patient portal access code [yes/no], and whether it was used [registered/not registered/cancelled account] was recorded for all patients in the sample by the EDW. The following functions were reported in this study: ordering prescription reauthorizations [yes/no], checking test results [yes/no], monitoring vital statistics [yes/no], and patient-physician messaging [yes/no]. Inspection of patient-initiated messages to their primary care physician suggested patients were contacting their physician directly to request prescription reauthorizations, rather than using the native function of the patient portal. Each-patient initiated message was therefore coded to reflect whether a prescription had been requested within the message. For the purposes of the prescription reauthorizations outcome, we created a single composite variable which represented whether a patient had requested a prescription reauthorization by either clicking the prescription button or through a secure message.

#### Health literacy

The Newest Vital Sign^31^ was assessed at the baseline interview. The Newest Vital Sign is one of the most common health literacy tests used.^32^ The assessment involves testing the ability to interpret information on a nutritional label of an ice cream container. Six questions are asked, and a single point is allocated for each correct answer. All of the information needed to identify the correct answer is available on the nutritional label, which can be inspected throughout the test. Scores are classified as limited (0–1), marginal (2–3), and adequate (4–6).

#### Participant characteristics

During the baseline interview, measures of age (55–59, 60–64, 65+), gender, race (White, African American, Other), and education (≤high school, some college or technical school, college graduate, graduate degree) were recorded. The following chronic conditions were also self-reported: arthritis, asthma, bronchitis or emphysema, cancer, coronary heart disease, depression, diabetes, heart failure, and hypertension. These data were grouped into 0, 1, and 2 or more chronic conditions.

### Data analysis

The chi-square statistic was used to determine group differences in who was offered a patient portal code during an appointment, and who subsequently registered for an account. People who registered but subsequently cancelled their accounts were removed due to small numbers (*n* = 6). Participant characteristics (age, gender, race, education, chronic conditions), and health literacy were entered into multivariable logistic regression analyses to predict the likelihood of being offered a patient portal code and subsequently registering it. A similar approach was taken to assess the likelihood of patient portal users using each of the available functions. Data were available on the proportion of patients receiving a prescription that needed a refill, and having a test result released onto the patient portal during the time that an individual patient’s portal was registered. These data were used to restrict the samples for analyses of these outcomes. The type I error rate was set at *P* < .05. SPSS version 22.0 was used for all analyses.

## RESULTS

### Sample

The baseline LitCog sample recruited 828 adults. Of these, 628 (75.8%) were recruited from the academic general internal medicine clinic where the patient portal was available. Ninety-four patients had not visited the hospital in the past 2 years and had not logged into the patient portal for the past 2 years. It is therefore likely that they were no longer patients at the hospital and they were excluded from analyses. The final sample for analyses was 534.

As shown in Table 1, patients were evenly distributed by age (55–59 years, 30.5%; 60–64 years, 30.7%; 65+ years, 38.8%). The majority were female (70.0%), White (65.2%), with a graduate degree (39.3%) and had 1 (34.1%) or 2 or more (48.5%) chronic conditions. Over half of the patients had an adequate level of health literacy (59.6%), with 23.0% and 17.4% classified as marginal and limited, respectively.

**Table 1:** Participant characteristics

	*n* (%)
Age	
55–59	163 (30.5)
60–64	164 (30.7)
65+	207 (38.8)
Gender	
Male	160 (30.0)
Female	374 (70.0)
Race	
White	345 (65.2)
African American	146 (27.6)
Other	38 (7.2)
Education	
≤High school	79 (14.8)
Some college or tech	111 (20.8)
College graduate	134 (25.1)
Graduate degree	210 (39.3)
Chronic conditions	
0	93 (17.4)
1	182 (34.1)
2+	259 (48.5)
Health literacy	
Limited	93 (17.4)
Marginal	123 (23.0)
Adequate	318 (59.6)

### Registration of the patient portal

Most patients (93.4%) had a patient portal access code generated for them. In univariable analyses, Whites (95.1%) and Other groups (94.7%) were more likely to be offered an access code than African Americans (89.0%; *P* = .046), although after controlling for age, gender, education, chronic conditions, this effect was eliminated (*P* > .05). Among patients who were offered an access code, 287 (57.5%) registered their accounts, 206 (41.3%) did not register their accounts, and 6 (1.2%) cancelled their accounts after registration. Cancelled accounts were removed from subsequent analysis.

In univariable chi-square analyses (Table 2), gender was a significant predictor of registering the patient portal (*P* = 0.034), with men more likely to register it than women (65.3% vs. 55.1%). Race was also a significant predictor of patient portal registration (*P* < .001), with Whites (71.7%) more likely to register their accounts than African American (27.7%) and “Other” races (41.7%). More educated people were more likely to register the patient portal (*P* < .001). For example, patients with a graduate degree were substantially more likely to register their accounts than those with a high school education or less (68.4% vs. 29.2%). Patients with fewer chronic conditions were more likely to register their patient portal (*P* = .001), with registration ranging from 70.9, 63.2, and 50.0% among those with 0, 1, and 2 or more chronic conditions, respectively. Patients with adequate health literacy (72.7%) were more likely than those with marginal (46.4%) or limited health literacy (21.7%) to have registered their patient portal account (*P* < .001).

**Table 2:** Univariable chi-square analyses predicting the likelihood of registering portal and using its functions

	Registration (%)	Messaging (%)	Prescription reauthorization (%)	Checking test results (%)	Vital statistics (%)
Age					
55–59	63.2	92.7	62.6	95.8	9.4
60–64	61.0	86.2	53.8	95.7	13.8
65+	51.9	89.7	48.5	95.8	9.3
Gender					
Male	65.3*	94.9*	60.8	96.9	14.3
Female	55.1	86.8	51.6	95.2	9.0
Race					
African American	27.7^‡^	83.3	55.6	82.9^‡^	11.1
White	71.7	91.8	54.3	98.2	11.7
Other	41.7	80.0	60.0	93.3	0.0
Education					
≤High school	29.2^‡^	76.2	30.0	95.2	4.8
Some college or tech	46.0	89.1	54.3	93.0	13.0
College graduate	68.8	88.4	51.8	94.2	7.0
Graduate degree	68.4	92.5	60.8	97.7	13.4
Chronic conditions					
0	70.9^†^	85.2*	46.3	98.3	11.5
1	63.2	95.4	54.2	97.1	10.2
2+	50.0	86.4	59.3	93.2	11.0
Health literacy					
Limited	21.7^‡^	61.1^‡^	38.9*	83.3*	11.1
Marginal	46.4	86.3	42.9	96.0	17.6
Adequate	72.7	92.7	59.0	96.7	9.2

Notes: *P < .05; ^†^*P* < .01; ^‡^*P* < .001; ns for outcomes [registration = 528]; [messaging = 287]; [reauthorizations = 279]; [results = 283]; [vital statistics = 287].

In multivariable analyses controlling for all participant characteristics, compared with African Americans, White patients were more likely to have registered their patient portal account (AOR, 4.19; 95% CI, 2.48-7.07; *P* < .001), but the ‘Other’ racial groups were no more likely (AOR, 2.14; 95% CI, 0.92-5.00; *P* = .077; Table 3). Compared with patients with a high school education or less, patients with a college graduate degree were more likely to have registered their patient portal account (AOR, 2.47; 95% CI, 1.19-5.13; *P* = .015). Patients with marginal or adequate health literacy were more likely to have registered their patient portal account than the limited health literacy group (AOR, 2.18; 95% CI, 1.06-4.49; *P* = .034; and AOR, 4.15; 95% CI, 2.07-8.35; *P* < .001, respectively). The effects of gender and chronic conditions were no longer significant in the multivariable model (*P* > .05).

**Table 3:** Multivariable logistic regression predicting the likelihood of registering portal and using its functions

	Registration AOR (95% CI)	Messaging AOR (95% CI)	Prescription reauthorization AOR (95% CI)	Checking test results AOR (95% CI)	Vital statistics AOR (95% CI)
Age					
55–59	1.69 (0.99–2.89)	1.17 (0.36–3.75)	1.86 (0.98–3.53)	0.83 (0.15–4.69)	1.38 (0.49–3.88)
60–64	1.22 (0.74–2.02)	0.45 (0.16–1.26)	1.13 (0.61–2.09)	1.06 (0.19–5.86)	1.96 (0.76–5.10)
65+	–	–	–	–	–
Gender					
Male	1.29 (0.81–2.07)	3.35 (1.06–10.57)*	1.46 (0.85–2.49)	0.98 (0.23–4.26)	1.66 (0.76–3.63)
Female	–	–	–	–	–
Race					
African American	–	–	–	–	–
White	4.19 (2.48–7.07) ^‡^	1.32 (0.39–4.43)	0.73 (0.32–1.66)	10.53 (2.14–51.76) ^†^	2.09 (0.61–7.17)
Other	2.14 (0.92–5.00)	1.49 (0.24–9.32)	1.74 (0.44–6.86)	3.22 (0.29–35.32)	N/A
Education					
≤High school	–	–	–	–	–
Some college or tech	1.60 (0.76–3.37)	1.80 (0.36–8.89)	3.89 (1.13–13.42)*	0.47 (0.03–6.38)	3.80 (0.38–38.12)
College graduate	2.47 (1.19–5.13)*	1.60 (0.37–6.99)	3.34 (1.03–10.91)*	0.24 (0.02–3.26)	2.11 (0.21–21.60)
Graduate degree	1.74 (0.86–3.53)	1.56 (0.36–6.80)	4.52 (1.40–14.61)*	0.42 (0.03–6.69)	4.20 (0.44–40.47)
Chronic conditions					
0	1.83 (0.99–3.37)	0.52 (0.19–1.47)	0.45 (0.22–0.91)*	2.38 (0.23–24.07)	0.92 (0.33–2.57)
1	1.32 (0.83–2.10)	2.02 (0.65–6.30)	0.67 (0.38–1.19)	1.66 (0.35–7.78)	0.84 (0.35–2.02)
2+	–	–	–	–	–
Health literacy					
Limited	–	–	–	–	–
Marginal	2.18 (1.06–4.49)*	3.23 (0.77–13.52)	1.09 (0.32–3.67)	4.74 (0.54–41.41)	0.95 (0.16–5.69)
Adequate	4.15 (2.07–8.35) ^‡^	7.78 (1.98–30.62) ^†^	2.00 (0.62–6.39)	3.43 (0.53–22.35)	0.34 (0.06–2.07)

Notes: *P < .05; ^†^*P* < .01; ^‡^*P* < .001; ns for outcomes [registration = 528]; [messaging = 287]; [reauthorization = 279]; [results = 283]; [vital statistics = 287].

^a^For the outcome “checking vital statistics,” the number in the “Other” race category was 0, and therefore an AOR could not be calculated. Analysis of this outcome therefore combined African American and “Other” to create a non-white race category. AOR = Adjusted Odds Ratio. All analyses are adjusted for age, gender, race, education, chronic conditions, and health literacy

### Use of the patient portal’s functions

#### Overall use

The median number of logins per month was 0.69, and this ranged from 0-9.35. The number of patients using 0, 1, 2, 3, and 4 patient portal functions was 3.5%, 6.3%, 35.2%, 48.4%, and 6.6%, respectively.

#### Patient to physician messaging

The majority of the sample (89.5%) had used the patient portal to send a message to their physician. There was an effect of health literacy, with the adequate health literacy group (92.7%) more likely to have used the messaging function than the marginal (86.3%) and limited (61.1%) health literacy groups (92.7%; *P* < .001). Men (94.9%) were more likely than women (86.8%) to have used the messaging function (*P* = .033), and patients with 1 chronic condition (95.4%) were more likely than those with no chronic conditions (0 = 85.2%) or 2 chronic conditions (86.4%; *P* = .042). In multivariable analyses, those with adequate health literacy (AOR, 7.78; 95% CI, 1.98-30.62; *P* = .003) and males (AOR, 3.35; 95% CI, 1.06-10.57; *P* = .040) were more likely to have used the message function.

#### Prescription reauthorizations

Most patients had received a prescription requiring a refill (97.2%) since registering their patient portal, and analyses for this outcome were restricted to this sample (*n* = 279). Over half of the sample (54.8%) had either used the prescription option on the patient portal, or had messaged their physician to request a reauthorization. In univariable analyses, patient with adequate health literacy (59.0%) were more likely to use the prescription function than those with marginal (42.9%) and limited (38.9%) health literacy (*P* = .046). In multivariable analysis, there was no effect for either health literacy category (*P* > .05; Table 3). More educated patients were consistently more likely to have used this function than those with a high school education or less (some college/tech: AOR, 3.89; 95% CI, 1.13-13.42; *P* = .032; college graduate: AOR, 3.34; 95% CI, 1.03-10.91; *P* = .045; and graduate degree: AOR, 4.52; 95% CI, 1.40-14.61; *P* = .012). Patients with no chronic conditions were less likely to have requested a reauthorization (AOR, 0.45; 95% CI, 0.22-0.91; *P* = .026).

#### Checking test results

The majority of patients had a laboratory test result released for them to view (98.6%) since registering their patient portal, and analyses for this outcome were restricted to this sample (*n* = 283). Most patients (95.8%) used the patient portal to check the results of a test. Univariable analyses suggested racial disparities, with White (98.2%) and Other groups (93.3%) more likely to have used this function than African American patients (82.9%; *P* < .001). Patients with adequate (96.7%) and marginal (96.0%) health literacy were more likely to have check test results than those with limited health literacy (83.3%; *P* = .025). Multivariable analysis indicated White patients were over 10 times more likely to have checked their test results than African American patients, although confidence intervals were wide (AOR, 10.53; 95% CI, 2.14-51.76; *P* = .004). Health literacy was not a significant predictor in the multivariable model (marginal: AOR, 4.74; 95% CI, 0.54-41.41; *P* > .05; adequate: AOR: 3.43; 95% CI, 0.53-22.35; *P* > .05).

#### Monitoring vital statistics

A minority of the sample (10.8%) had checked their vital statistics via the patient portal. In both univariable and multivariable analyses, there were no significant predictors of using this function (*P* > .05).

## DISCUSSION

In this sample of older American adults recruited from an urban general internal medicine ambulatory care clinic, we linked cohort data to information available on patients’ registration and subsequent use of an online patient portal. Despite the majority of the cohort being offered the opportunity to register their patient portal, a little over half decided to complete this task. There were stark racial, health literacy, and educational disparities in patient portal registration, even after adjustment in multivariable models. For example, 22% of patients with limited health literacy registered their patient portal accounts compared with 73% of those with adequate health literacy skills. A similar magnitude of difference was observed between races and educational levels. In contrast to existing research among the general adult population, in this sample of older adults healthier patients were more likely to have registered their patient portal than those reporting chronic conditions.^4^

There were fewer disparities in the use of the patient portal’s functions, but some observations have important clinical consequences. Highly educated patients were consistently more likely to use the patient portal for prescription refill requests. Alternative methods are available, but they can be more time consuming for the patient and cannot be used when the clinic is closed. The more frequent use of this function by more educated patients may facilitate more timely prescription renewal, resulting in disparities in medication adherence.^33^^,^^34^ For example, in a large observation cohort of diabetic patients, nonuse of the refill function on an online patient portal was associated with poorer adherence to statins.^35^

An equally concerning observation was that White patients were over 10 times more likely to have checked test results online. Currently within the study practice, there are no formal processes to ensure that patients with online test results review them. It is possible to manually view each patient’s record and determine whether online test results have been read, but this process remains cumbersome and variably employed. Patients who do not review their test results may therefore be at risk of adverse health outcomes if clinical instructions contained within their release are missed. Adequate safety measures, such as an automatic system for alerting physicians that a test result has not been inspected, should be put in place to minimize potential adverse events, especially those that disproportionately affect African American patients.

Patients with an adequate level of health literacy were nearly 8 times more likely than those with limited health literacy to use the secure patient–physician messaging function. Inspection of the messages among this cohort suggested patients used this function to update their provider (e.g., appointments with other clinicians) and ask advice about chronic and acute health problems (e.g., referrals or self-care tasks). The providers of low health literacy patients may therefore receive fewer updates about their health, and they may be less informed about their patient’s wellbeing prior to an appointment. Patient–physician dialogue is an important part of patient engagement, and this finding suggests that communication inequalities may be created through the implementation of patient portals.^36^ Health literacy disparities may be particularly important among older populations as this patient group are at greater risk of having inadequate basic skills.^27^^,^^28^

Broadly, our findings suggest research should focus on addressing the barriers faced by older underserved populations at the registration stage of patient portal initiation. There is an established “digital divide” in Internet connectivity,^37^ and this is likely to be a contributing factor.^4^^,^^38^ National data indicate that while nearly 90% of the population uses the Internet, levels are lower among older groups (age 65+, 57%), African Americans (81%), and Hispanics (83%).^37^ Estimates for educational differences range from 76% among those with a high school education or less up to 97% among those with a Bachelor’s degree or more. In 2009, nine of Chicago’s 76 neighborhood communities had <50% home connectivity, and 19 had <65%.^39^ Healthcare systems adopting online technologies such as patient portals should continuously monitor Internet connectivity rates within their population to avoid exacerbating disparities. This may be particularly relevant to healthcare systems who serve predominantly older patient groups. Attention should be paid to home Internet connectivity, as patients may have access at a less convenient location which would inhibit regular patient portal use. It should be noted that the digital divide is less pronounced when considering smartphones,^40^ suggesting that patient portals should have a smartphone interface to allow access from multiple devices.

Additional barriers faced by patients include difficulties with usability,^9^^,^^15–18^ forgetting to enroll after being provided with an access code,^41^ a lack of interest in the functions available,^41^ and negative attitudes toward technology.^41^^,^^42^ While trust can be a barrier to sharing electronic health data,^43^ this is not always supported by patient-reported data.^15^^,^^41^^,^^44^^,^^45^ These factors could be particularly relevant to older patient groups who have less experience with information technology.^37^ There is also some evidence to suggest disparities in barriers to patient portal registration and use,^41^ but further work is needed to identify the underlying causes of inequalities in patient portal use. Our finding that lower health literate patients were less likely to use the messaging function may indicate that they lack the ability or confidence to compose written communications, or have lower levels of awareness that this function exists. The lower levels of result checking among African American patients suggests a similar barrier, and physicians should ensure that all patients are aware of how they will be notified about medical test outcomes, and provide alternatives if the patient portal is unsuitable. User-testing and improving functionality are important elements of introducing patient portals, and may help to ameliorate disparities.

Most patients in this sample had messaged their physician (90%), checked a test result (96%), and requested a reauthorization of a prescription (55%), but few monitored their vital statistics (11%). The low prevalence of vital statistic monitoring suggests that if patients are to become more effective consumers in the healthcare environment, promotion of the availability of such functions may be needed. The most comprehensive review in the area concluded that health outcomes are only likely to be affected if patient portals are offered in tandem with active case management such as in-person visits and healthcare provider monitoring.^4^ There are an array of online health technologies available in today’s marketplace,^46–49^ and if patient portals are to keep pace, more comprehensive tools may be needed to attract users.

This study has limitations that should be acknowledged. We were unable to control for Internet connectivity; it is therefore unclear if disparities in registration indicated a lack of opportunity or interest. Levels of social support were not recorded, which may be an important mechanism for bridging the digital divide between health literacy groups.^21^ The concept of eHealth literacy may also be important to navigating an online patient portal,^50^ but we were unable to record the construct here. After restricting the sample to investigate differences in use of the patient portal’s functions, sample sizes for minority groups were small. This is likely to have compromised our ability to detect study effects, and resulted in wide confidence intervals for some estimates. These data were collected from a single institution, and findings should be replicated at other sites and with other patient portals. Data were also from a sub-set of the LitCog sample, as no patient portal was available at the Federally Qualified Health Centers used for patient recruitment. As a result, education and literacy levels were high and we may have underestimated the magnitude of disparities that would occur if this technology was rolled out among more deprived populations. Although we controlled for the number of chronic conditions, we were unable to account for the broad array of reasons that a patient might log on to the patient portal and use its functions. However, investigations related to using the prescription reauthorization and test result functions were restricted to include only those for whom the outcome was relevant.

## CONCLUSION

The introduction of patient portals is widespread and disparities in their registration and subsequent use are being documented. This study is among the first to demonstrate that objectively measured health literacy is associated with registering a patient portal account, and the magnitude of this effect was similar to that of race. More educated patients were also more likely to register their accounts. There were fewer notable disparities in the use of patient portal functions; however, literacy disparities in secure messaging, educational differences in prescription ordering, and race inequalities in checking test results are all cause for concern.

## CONTRIBUTORS

All authors have contributed to writing the manuscript and review of the final text. Dr Smith and Dr Wolf act as guarantors for the content. Specific contributions are as follows:

Planning: Smith, Wolf, O’Conor, Goel

Conduct, data extraction, and analysis: Smith, O’Conor, Curtis, Aitken, and Goel

Reporting: Smith, O’Conor, Aitken, Curtis, Goel, Wolf

## COMPETING INTERESTS

We report no conflict of interests.

## FUNDING

Financial disclosure: This project was supported by the National Institute on Aging (R01 AG030611), the National Center for Research Resources (5UL1RR025741), and the National Center for Advancing Translational Sciences (Grant 8UL1TR000150). The content is solely the responsibility of the authors and does not necessarily represent the official views of the National Institutes of Health. Smith is currently supported by a Cancer Research UK Fellowship.
